# High Steroid Sensitivity among Children with Nephrotic Syndrome in Southwestern Nigeria

**DOI:** 10.1155/2014/350640

**Published:** 2014-07-16

**Authors:** Taiwo Augustina Ladapo, Christopher Imokhuede Esezobor, Foluso Ebun Lesi

**Affiliations:** ^1^Department of Paediatrics, College of Medicine, University of Lagos, PMB 12003, Lagos, Nigeria; ^2^Department of Paediatrics, Lagos University Teaching Hospital, Idi-Araba, PMB 12003, Lagos, Nigeria

## Abstract

Recent reports from both Caucasian and black populations suggest changes in steroid responsiveness of childhood nephrotic syndrome. This study was therefore undertaken to determine the features and steroid sensitivity pattern of a cohort of black children with nephrotic syndrome. Records of children managed for nephrotic syndrome from January 2008 to April 2013 were reviewed. Details including age, response to treatment, and renal histology were analysed. There were 108 children (median age: 5.9 years, peak: 1-2 years), 90.2% of whom had idiopathic nephrotic syndrome. Steroid sensitivity was 82.8% among children with idiopathic nephrotic syndrome but 75.9% overall. Median time to remission was 7 days. Median age was significantly lower in steroid sensitive compared with resistant patients. The predominant histologic finding in resistant cases was focal segmental glomerulosclerosis (53.3%). No cases of quartan malaria nephropathy or hepatitis B virus nephropathy were diagnosed. Overall mortality was 6.5%. In conclusion, unusually high steroid sensitivity is reported among a cohort of black children. This is likely attributable to the lower age structure of our cohort as well as possible changing epidemiology of some other childhood diseases. Surveillance of the epidemiology of childhood nephrotic syndrome and corresponding modifications in practice are therefore recommended.

## 1. Introduction

Childhood nephrotic syndrome (NS) is the commonest glomerular lesion encountered in childhood [1, 2]. Although various histological features have been described, the most important determinant of outcome of this condition is steroid responsiveness which is, however, not uniformly distributed globally. High steroid responsiveness has traditionally been demonstrated in temperate regions of the world and, conversely, high steroid resistance in tropical regions of the world like Nigeria [3–10]. Consequently, the black race is often considered an indication for kidney biopsy in children with NS.

Recent reports however suggest changes in steroid responsiveness of nephrotic syndrome, with steroid resistance being increasingly reported in non-blacks while some regions have experienced increasing steroid sensitivity [11–15]. Previous reports from Nigeria demonstrated high steroid resistance ranging between 35% and 92% [6–9, 15–18]. Cursory observations of the cohort of children receiving care in our centre suggested high steroid sensitivity, hence the need for this report. This study therefore evaluates the pattern of steroid sensitivity among a cohort of black children with childhood nephrotic syndrome.

## 2. Materials and Methods

The study was conducted at the Paediatric Nephrology Unit of the Lagos University Teaching Hospital, a 760-bed tertiary hospital in Southwest Nigeria. The hospital is one of the two referral centres in the state providing renal care to children in Lagos State and its environs. It therefore caters for children across all socioeconomic strata. The records of all children managed for nephrotic syndrome between January 2008 and April 2013 were reviewed. Nephrotic syndrome was diagnosed based on the following: 24-hour urine protein > 40 mg/m^2^/hr or spot urine protein: creatinine ratio > 200 mg/mmol, hypoalbuminemia (serum albumin < 25 g/L), generalized oedema, and hypercholesterolemia (serum cholesterol > 5.2 mmol/L) [1, 2].

The following results were retrieved: blood pressure, urine microscopy and culture, serum electrolytes, calcium, phosphate, urea and creatinine, blood film for malaria parasite, haemoglobin genotype, renal ultrasound scan and screening for hepatitis B, hepatitis C, and human immunodeficiency virus, antinuclear antibodies, ANCA, and Complement levels. Microscopic haematuria was defined as >5 red blood cells (RBCs) per high field of a centrifuged urine specimen and glomerular filtration rate (eGFR) was estimated using the modified Schwartz formula. Chronic disease was defined according to the Kidney Disease Outcomes Qualitative Initiative (KDOQI) guidelines [19]. Hypertension was defined as blood pressure > 95th centile for age, gender, and height on three consecutive occasions [20]. Urinary tract infection was diagnosed in the presence of supportive urinalysis findings and significant growth of uropathogenic organism in an appropriately collected urine specimen [2]. Systemic lupus erythematosus (SLE) was diagnosed using the revised American College of Rheumatology criteria [21]. Socioeconomic classification was done using the classification by Oyedeji [22] which employs the educational status and occupation of parents.

## 3. Treatment Regimen

At first presentation, patients receive oral prednisolone at 60 mg/m^2^ daily for 4–6 weeks. Since 2012, following KDIGO (Kidney Disease Improving Global Outcomes) recommendations, we have extended treatment to 8 weeks to define steroid resistance [23]. Following remission, the dose is reduced to 40 mg/m^2^ on alternate days for 4 weeks and gradually tapered over 3–5 months. Steroid dependence (SD) was treated with the addition of levamisole (2.5 mg/kg/day) on alternate days or cyclophosphamide (oral or intravenous). Steroid resistance was treated with oral (2 mg/kg/day for 8weeks) or intravenous (500 mg/m^2^/month for 6 months) cyclophosphamide, angiotensin converting enzyme (ACE) inhibitors, or cyclosporine. Since 2012, cyclosporine rather than cyclophosphamide has become the preferred drug for the management of steroid resistance [23]. Renal biopsies were performed by the paediatric nephrologists for steroid resistant or secondary cases of NS. Tissue was considered adequate for reporting if at least 5 glomeruli were present.

## 4. Definition of Terms [1, 2]

Terms are defined as follows: remission: nil or trace proteinuria < 30 mg/dL for 3 consecutive days after commencing treatment; steroid resistant nephrotic syndrome (SRNS): failure to achieve remission after 6–8 weeks of daily prednisolone; relapse: recurrence of 100 mg/dL (≥2+) proteinuria for ≥3 consecutive days after having been in remission; steroid dependent nephrotic syndrome (SDNS): two consecutive relapses during alternate day steroid therapy or within 14 days after cessation of steroids; frequently relapsing nephrotic syndrome (FRNS): two or more relapses within 6 months of initial response or ≥4 relapses in any 12-month period.

## 5. Statistical Analysis

Data was analysed using the Statistical Package for Social Sciences software version 20. Continuous data were represented as mean or median while categorical data were presented as percentages. Significance between steroid sensitivity and some variables was determined using chi-square test while comparison of means was done with Student's *t*-test. A *P* value of <0.05 was considered statistically significant.

## 6. Results

**Figure 1:**
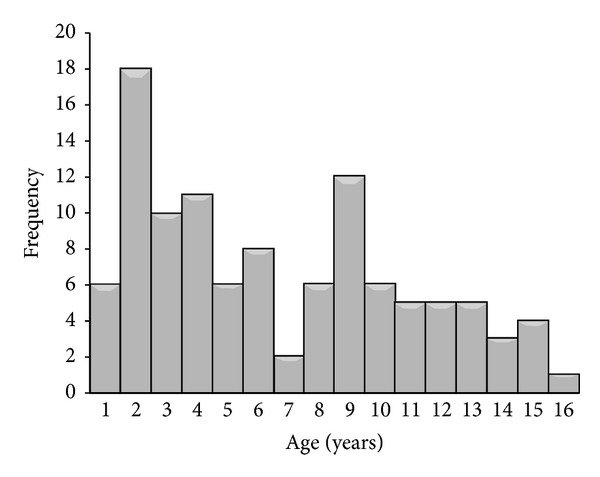
Age distribution of 108 patients with nephrotic syndrome.

We managed 108 children, 68 males and 40 females (m : f = 1.7 : 1). Age range was 8 months to 15.4 years (median 5.9 years). Their age distribution is shown in Figure 1, reflecting a bimodal distribution with a peak at 1 to 2 years and a slightly lesser one at about 9-10 years. About half, 53 (49.1%), were aged less than ≤5 years. Of 88 children with complete data on socioeconomic status, majority, 38 (45.2%), were from the lower socioeconomic class while 31 (36.9%) and 15 (17.9%) were from the middle and upper socioeconomic classes, respectively. Mean serum albumin was 2.05 g/L ± 0.82 with mean serum cholesterol of 10.4 ± 4.4 mmol/L. Most of the children, 94 (87%), had normal creatinine levels at presentation. Haematuria was present in (48) 44%, hypertension in (46) 43%, while (27) 25% had urinary tract infection.

### 6.1. Aetiology and Steroid Response

**Figure 2:**
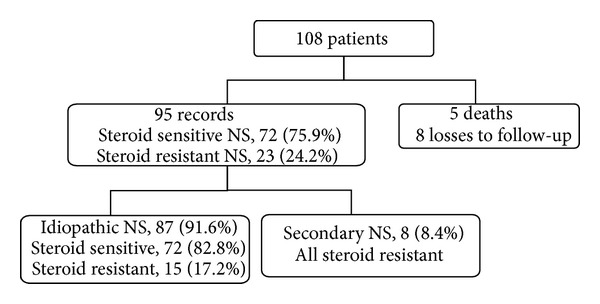
Flow chart of steroid sensitivity of patients with nephrotic syndrome.

Records on aetiology were available for 102 patients. Of these, the majority (92; 90.2%) were idiopathic. A secondary cause was found in 10 children as follows: systemic lupus erythematosus (3), sickle cell disease (3), chronic glomerulonephritis (2), infantile NS (1) and Down's syndrome with cyanotic glomerulopathy (1). Steroid sensitivity pattern was available for 95 children (Figure 2). Five died before steroid sensitivity could be determined while others were unavailable due to loss to follow-up. Steroid sensitivity among children with INS was 82.8% (72/87) but 75.9% (72/95) overall. Twelve (16.7%) of those with SSNS were either steroid dependent or frequently relapsing, and 23 children (24.2%) were steroid resistant. Time to remission in steroid sensitive patients ranged from 3 to 38 days (median 7 days).

Fifteen children were biopsied: 14 with SRNS of whom 8 had idiopathic SRNS and an 11-year-old male at presentation on account of age, macroscopic haematuria, and hypertension. Biopsy was not done in the others due to refusal to consent, transfer to another facility, or death. Histological findings were as follows: focal segmental glomerulosclerosis (FSGS) in 8 (53.3%), minimal change disease (MCNS) in 3 (20%) and 1 (6.7%) each of membranous nephropathy, membranoproliferative glomerulonephritis (MPGN), diffuse proliferative glomerulonephritis, and class 3 lupus nephritis. Histologic patterns among the 8 with idiopathic SRNS were FSGS in 5 (62.5%), MCNS in 2 (25%), and MPGN in 1 (12.5%).

Table 1 shows a comparison of variables between the steroid resistant and responsive groups. Lower serum albumin, higher serum cholesterol, older age, haematuria, and raised serum creatinine were all associated with steroid resistance. Only association with albumin and UTI did not reach statistical significance.

### 6.2. Outcome

Seven children (6.5%), all with SRNS, developed chronic kidney disease (CKD) of whom 5 progressed to end-stage kidney disease (ESKD) during the review period. One was with haemoglobin SS disease (HBSS) presented in CKD while the others (SLE: 2; FSGS: 2; idiopathic: 2) developed CKD within 2 years of diagnosis. There were 7 deaths as follows: complications of ESKD in 3 (lupus nephritis: 1, FSGS: 1, and INS: 1); AKI in 2 patients, one of whom had lupus nephritis, and 2 newly diagnosed patients with undetermined steroid sensitivity one of whom died from a cerebrovascular accident. Overall mortality was therefore 6.5% but 21.8% in those with SRNS.

## 7. Discussion

Our study revealed high prevalence of steroid sensitivity among a cohort of black children with NS. In summary about 76% of the whole cohort and approximately 83% of the cohort with idiopathic childhood nephrotic syndrome achieved remission following treatment with steroids. The high proportion of steroid sensitivity in this study is remarkable because such high sensitivity has rarely been described in a large sample of black children and ranks similar to that described among children of other races [2–4]. Various studies describe NS in children of African descent as being predominantly steroid resistant and nonminimal change on histology. In Nigeria, steroid resistance ranges between 35% and 92% among different geographical locations [6–9, 15–17] Table 2, higher than the 23% in the current study. Doe et al. in Ghana [24] reported steroid resistance of 50% amongst their cohort while Bhimma's group in South Africa [25] reported about 86% steroid resistance among black children. In the latter study, steroid sensitivity among Indian children in the same cohort was over 65% again highlighting racial differences in steroid sensitivity. This has led to the recommendation of a kidney biopsy as part of the initial evaluation of black children with NS.

The reasons for this striking observation of high steroid sensitivity likely include the younger age structure of our cohort. The statistically significant lower median age of the steroid sensitive compared with steroid resistant patients supports this. A small study from southern Nigeria [14] that reported 80% steroid sensitivity had a young population with a mean age of 5.8 years. Asinobi's group [13] also from Nigeria reported higher steroid sensitivity but their cohort comprised only children aged ≤ 5 years. This age pattern is similar to that reported among Caucasians and Asian children where high steroid sensitivity has been reported [1–4]. For instance, in a report from the United Kingdom [3], median age of children with SSNS was 4.5 years compared with 6 years in those with SRNS. We therefore argue that age and race should be considered together as strong predictors of response to steroid rather than the sole reliance on race.

Another plausible explanation for the increased sensitivity observed in our cohort is the absence of certain previously prominent secondary aetiologies of NS in our environment resulting in a relatively higher idiopathic pool, the majority of whom were steroid sensitive. This is a likely consequence of intensified efforts by the Government to reduce the burden of some childhood conditions in the region. For instance, we did not diagnose any case of quartan malaria nephropathy (QMN), which was previously reported to be a leading cause of steroid resistant nephrotic syndrome in our environment [26]. Previous Nigerian authors [8, 27] have similarly alluded to the reduced incidence of QMN in the region. Malaria control programs have resulted in improved access to antimalarial drugs over the last two decades and this is likely to have contributed to the decline in associated nephropathy. Doe's group in Ghana [2] also found no evidence of a tropical form of nephrotic syndrome in their patients. In the same vein, in contrast with reports from South Africa [5] and Ghana [24], we did not diagnose infections such as hepatitis and schistosomiasis, which we attribute to improved vaccination rates and access to portable water, respectively. These diseases have seemingly been replaced by others like SLE and sickle cell anaemia, as also observed by Olowu et al. [27]. Recent availability of diagnostic facilities for the former and improved survival beyond early childhood for the latter could account for this observation.

Contrariwise, on the global scene, the incidence of steroid resistant NS in various parts of the world such as the USA [11, 28], Poland [12], India [13], and Canada [29] is reportedly on increase which has been linked to increasing FSGS among these populations. Although Kim's group [11] and Bonilla-Felix et al. [28] attributed their findings to the large proportion of the African-Africans in their cohort, similar observations in homogenous or near homogenous Caucasian populations [12, 28] suggest the role of other factors. The reasons for these changes however remain of continued research interest [30].

Socioeconomic class is thought to play a role in NS although the exact associations are not very clear. The majority of our patients were from the lower socioeconomic class similar to a report from India [31] where 84% of study participants were from the lower socioeconomic class. While this may suggest a role for infections which are more prevalent in this socioeconomic class of children, we did not find a high prevalence of infection associated NS in this study. We however also reported a significant proportion of children from the middle socioeconomic class suggesting the role of other factors which are not apparent from this study. The aetiology of INS remains of continued research interest globally with extensive research into genetic mechanisms howbeit largely in the developed world.

FSGS was also our predominant histologic finding, a finding consistent with other African studies [16, 24, 25], especially since the era when kidney biopsy was reserved commonly for those with clinical and treatment features not in keeping with minimal change disease. Since minimal change NS is predominantly steroid sensitive, it is safe to assume that if all the children in the present study were to undergo kidney biopsies, it would have been the most predominant histological pattern. Higher cholesterol levels, hypertension, and haematuria were associated with increased steroid resistance, consistent with published data [32]. Our findings also support the reportedly high frequency of UTI in NS, particularly SRNS [33], and emphasize the need for screening for this infection in affected children.

As expected, progression to CKD and mortality were predominantly a function of with steroid resistance [1, 4, 7]; hence, our lower overall mortality of 6.5%, compared with 6.6–14.3% in other African reports [7, 16, 24], is not surprising. Mortality was particularly high among those with steroid resistant nephrotic syndrome who progressed to ESKD. This is largely a consequence of a combination of factors which include high cost of care, inadequate facilities for long-term dialysis, and lack of transplant facilities. Late presentation, hence advanced disease in two patients one of whom presented with a cerebrovascular accident, also contributed to the high mortality reported.

In conclusion, our study reports high steroid sensitivity in a large group of black children. Although its retrospective nature resulted in incomplete data in some cases, this was compensated for by its large sample size. This study and few others in the region [14, 15] suggest a changing pattern of steroid sensitivity of childhood NS which may reflect an adaptation to the changing epidemiology of some childhood diseases as alluded to by previous authors. Surveillance of the epidemiology of childhood NS and corresponding modifications in practice guidelines over time are therefore recommended.

## Figures and Tables

**Table 1:** Comparison of variables between steroid responsive and nonresponsive groups.

	SSNS	SRNS	*P*
Mean age (years)	5.8 ± 3.9 (median 5)	8.8 ± 4.8 (median 10.1)	0.004
Age group (years/number)			0.01
0–5 (48)	41 (85.4%)	7 (14.6%)	
6–10 (27)	20 (74.1%)	7 (25.9%)	
>10 (20)	10 (50%)	10 (50%)	
Serum albumin	2.1 ± 0.8	1.9 ± 0.8	0.57
Serum cholesterol	9.8 ± 4.0	12.6 ± 5.0	0.03
Hypertension∗ (89)	21/62 (33.9%)	16/23 (69.6%)	0.006
Haematuria∗ (95)	25/72 (36.2%)	12/23 (60%)	<0.001
UTI∗ (89)	15 (22.7%)	7 (35.0%)	0.32

Raised creatinine	2/72 (2.7%)	11/23 (47.8%)	<0.001

UTI: urinary tract infection. *Indicates number available for review for variable; percentages are of the total within the group.

**Table 2:** Steroid responsiveness of nephrotic syndrome across Nigeria.

Authors/year of publication	Region of Nigeria	Total No (mean/median age) years	SSNS, N (%)
F. U. Eke and N. N. Eke (1994) [6]	Port-Harcourt, south-south	102 (—)	23 (22.5)
Ibadin and Abiodun (1998) [7]	Benin, south-south	58 (8.2 ± 0.5)	30 (51.7)
Asinobi et al. (1999) [8]	Ibadan, south-west	41 (—)	3 (8.0)
Okoro and Okafor (1999) [9]	Enugu, south-east	346 (5–7)	104 (30)
∗Adedoyin et al. (2001) [16]	Ilorin, north-central	17 (8.8)	3 (17.6)
Ibadin and Ofovwe (2003) [18]	Benin, south-south	51 (5)	35 (68.6)
Asinobi et al. (2005) [15]	Ibadan, south-west	20 (4.0)	12 (60)
^*χ*^Anochie et al. (2006) [14]	Port-Harcourt, south-south	20 (5.8 ± 3.8)	16 (80)
^*χ*^Olowu et al. (2010) [17]	Ile-Ife, south-west	42 (9.95 ± 3.15)	19 (45.2)
Current study	Lagos, south-west	108 (6.6 ± 4.2/5.9)	72 (75.9) ^*χ*^72 (82.8)

**N**ote: ∗8 defaulted, ^*χ*^idiopathic nephrotic syndrome only, (—) data not given.
